# Arthroscopic Management of Anterior Shoulder Instability: A Study of Functional Outcome and Analysis

**DOI:** 10.7759/cureus.61870

**Published:** 2024-06-07

**Authors:** Raja Ramesh Badavath, Thirlapuram Sandeep Kumar, Lalithmohan Chodavarapu

**Affiliations:** 1 Department of Orthopedics, Nizam's Institute of Medical Sciences, Hyderabad, IND

**Keywords:** external rotation, remplissage, hill-sachs lesion, bankart lesion repair, anterior shoulder instability

## Abstract

Background

The remarkable range of motion of the shoulder comes at the cost of increased instability, especially anterior instability. Arthroscopic Bankart repair with or without remplissage, which is a minimally invasive surgery, is the preferred treatment for recurrent anterior instability. This study investigated the effectiveness of Bankart repair, with or without remplissage, in restoring function, preventing redislocation, and improving patient satisfaction.

Methods

A prospective observational study examined 40 patients (19-50 years old) with recurrent anterior instability and MRI-confirmed Bankart or Bankart with Hill-Sachs lesions. Patients underwent arthroscopic Bankart repair with or without remplissage based on the inclusion criteria of this study. Preoperative assessments included demographics, history, physical examination, American Shoulder and Elbow Surgeons (ASES) score, Quick Disabilities of the Arm, Shoulder, and Hand (DASH) score, ROWE score, and plain MRI of the shoulder joint. Post-operative radiographs and rehabilitation were advised. Functional recovery was evaluated at three months and six months after surgery.

Results

All patients underwent Bankart repair. Among them, 22 with engaging Hill-Sachs lesions received an additional remplissage procedure. Both groups showed significant improvements in their functional scores (p<0.05) and returned to their prior activities. However, the additional remplissage group had a slightly reduced mean external rotation (86.59°) compared with the Bankart repair-only group (90°). Notably, the recurrence rate was very low, with only one patient (2.5%) experiencing instability.

Conclusion

Our study emphasizes the importance of proper capsulolabral tissue elevation to achieve a sufficient labral bump during Bankart repair. This technique allowed us to efficiently use only two suture anchors in 35 cases (87.5%). Additionally, remplissage was performed on all identified engaging Hill-Sachs lesions. We found that proper anchor placement and suturing techniques were crucial for successful Bankart repair. The emphasis on the potential cost benefits of a two-anchor approach is a valuable contribution to the field.

## Introduction

The shoulder joint is the most commonly dislocated joint in the body, accounting for nearly half of all dislocations and affecting roughly 2% of the general population [1,2]. Anterior instability is the most frequent type, often caused by trauma [3]. Recurrent anterior instability typically involves soft tissue damage, including a Bankart lesion (tearing of the anteroinferior capsular-labral complex from the glenoid) and a Hill-Sachs lesion (a depression fracture on the posterior-superior aspect of the humeral head caused by impaction with the anterior glenoid rim) [4,5].

Several arthroscopic techniques have been developed for shoulder stabilization. These procedures primarily focus on reconstructing the capsular-labral complex using sutures and anchors placed within the joint and secured to the glenoid [6,7]. Arthroscopic Bankart repair has become the gold standard for treating recurrent anterior instability. It offers significant advantages over traditional open Bankart repair, including reduced post-operative pain, blood loss, and limitations in external rotation. Additionally, it minimizes the risk of complications like glenoid fractures [8-10]. The arthroscopic approach is also less time-consuming and allows for better post-operative range of motion [11,12]. This study aimed to evaluate the effectiveness of arthroscopic Bankart repair, with or without remplissage, in restoring shoulder function, preventing redislocation, and improving patient satisfaction in individuals with anterior shoulder instability.

## Materials and methods

This prospective, observational study examined 40 patients with recurrent anterior shoulder instability at Nizam's Institute of Medical Sciences, Hyderabad, India, for Arthroscopic Bankart repair with or without remplissage between April 2021 and March 2022. The study was approved by the Institutional Ethics Committee of Nizam’s Institute of Medical Sciences (approval number: EC/NIMS/2850/2021). Written informed consent was taken from all participants.

Inclusion criteria were age 18-64 years, recurrent anterior instability history (>one dislocation), MRI-confirmed Bankart lesion, and Hill-Sachs lesion. Exclusion criteria were first-time dislocation, age ≥65 years, Bankart lesion with >25% glenoid bone loss on MRI, voluntary dislocators, psychiatric disorders, neuropathic joint, unfitness for anesthesia, and refusal of consent.

**Figure 1:**
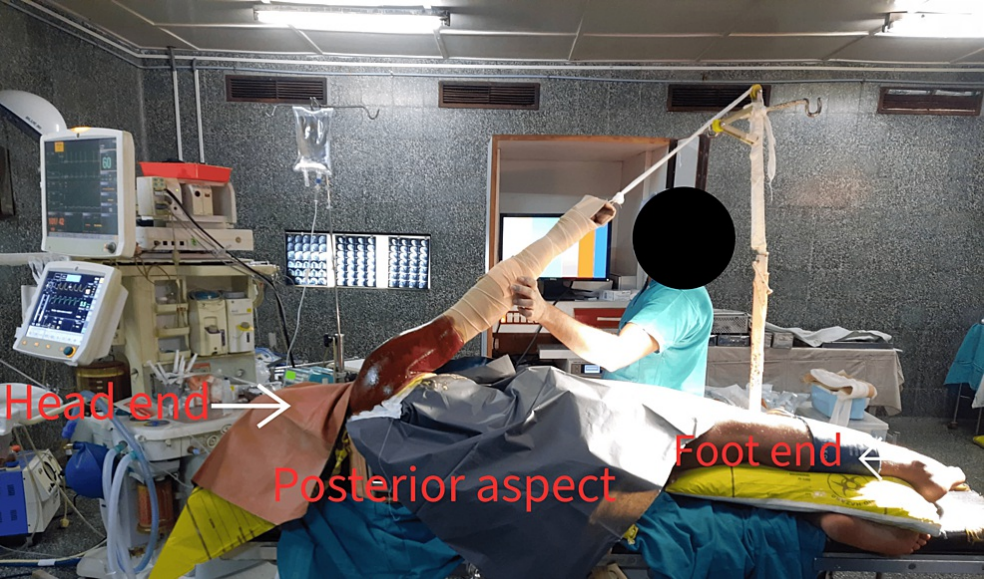
Lateral decubitus position.

Pre-operative evaluation included demographic data, detailed history, and a comprehensive physical examination were performed. This included age, sex, involved side, mode of injury at first dislocation, and number of prior dislocations. Assessment of the shoulder by inspection, palpation, range of motion, Jobe relocation release test, and apprehension test. Routine blood tests, standard shoulder radiographs (anteroposterior and axillary views), and plain magnetic resonance imaging of the affected shoulder were obtained. Pre-operative functional scoring was performed using the American Shoulder and Elbow Surgeons (ASES) score [13], Quick Disabilities of the Arm, Shoulder, and Hand (DASH) score [14], and ROWE score to establish a baseline [15]. A single surgeon performed all surgeries under general anesthesia. Patients were positioned in lateral decubitus position with the affected shoulder abducted at 40 degrees and forward flexion at 20 degrees (Figure 1).

**Figure 2:**
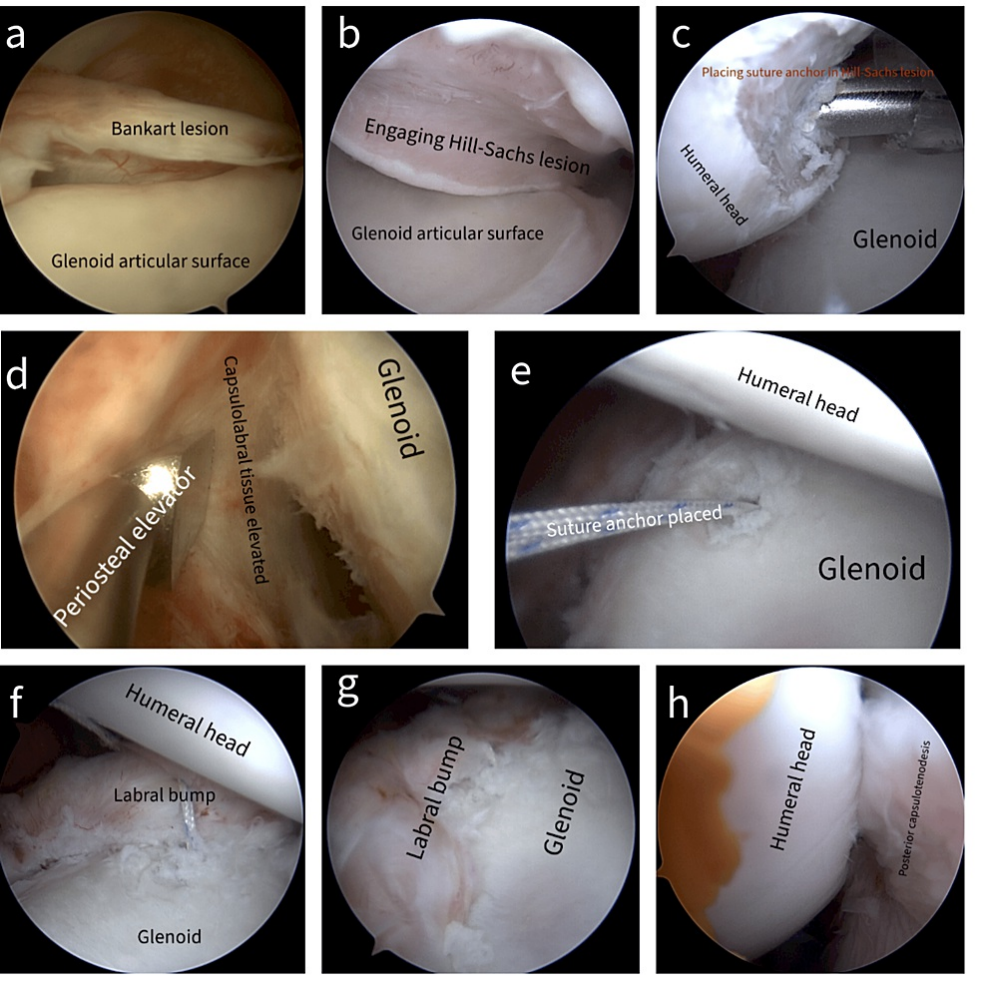
Arthroscopic images showing Bankart repair and remplissage. (a) Viewing Bankart lesion through posterior portal, (b) viewing engaging Hill-Sachs lesion through posterior portal, (c) viewing through anterosuperior portal showing suture anchor placement in Hill-Sachs lesion, (d) viewing capsulolabral tissue elevation through anterosuperior portal, (e) first suture anchor placed at anteroinferior rim of glenoid articular surface, (f) labral bump created with one suture anchor, (g) labral bump achieved with two suture anchors, and (h) viewing posterior capsulotenodesis into Hill-Sachs lesion through anterosuperior portal.

The three following standard portals were created to access the shoulder joint: posterior, anterosuperior, and anteroinferior. Diagnostic arthroscopy was performed to confirm the Bankart lesion and to assess the size of the Hill-Sachs lesion (Figures 2a-2h). For engaging Hill-Sachs lesions, a 5 mm suture anchor was placed near the articular cartilage to prepare for remplissage later. The torn labrum (Bankart lesion) was carefully detached from the glenoid using a periosteal elevator. The bone surface was roughened to promote healing. The labrum was completely freed before anchor placement. The first suture anchor was typically positioned at the inferior aspect (5:30-6 o'clock position) of the glenoid, 2-3 mm on the articular surface from the edge through the anteroinferior portal. A suture passer was used to shuttle the suture and tie a knot on the capsular side of the labrum, effectively pushing the labrum back onto the glenoid and creating a "labral bump" for stability. One or two additional anchors were placed superiorly, recreating the labral bump with each anchor. To address the Hill-Sachs lesion, the arthroscope was switched to the anterosuperior portal. Sutures from the anchors were tied in a mattress configuration over the Hill-Sachs lesion, creating a posterior capsulotenodesis to fill the defect and improve stability. With the arthroscope back in the posterior portal, checked the quality of both the Bankart repair and remplissage.

The operated shoulder was immobilized in a sling for the first three weeks. Gentle pendulum exercises and forward shoulder elevation up to 90 degrees were permitted from weeks three to six to encourage controlled movement. After week six, patients could initiate exercises targeting a full range of motion in the shoulder joint. Patients were monitored at regular intervals to assess their progress. During follow-up visits, shoulder range of motion and functional outcome scores (ASES, Quick DASH, and Rowe scores) were evaluated. The results were presented as frequencies. Statistical analysis was performed using IBM SPSS Statistics version 20.0 software (Chicago, IL: IBM Corp.). A p-value of less than 0.05 was considered statistically significant.

## Results

Age group

In this study, half (50%, n=20) of the participants fell within the 19-25 years age group. Another 30% (n=12) were between 26 and 35 years of age, and the remaining 20% (n=8) were in the 36-50 age range (Table 1).

**Table 1:** Age distribution of study participants (n=40). Data have been presented as number of participants (n) and percentage.

Age group (years)	Frequency (n)	Percentage
19-25	20	50%
26-35	12	30%
36-50	8	20%
Total	40	100%

Sex distribution

The study had a significant gender imbalance. An overwhelming majority of the participants, nearly all at 97.5% (n=39), were male. There was very limited female participation, with only one female participant which constitutes a mere 2.5% (n=1) of the total study population (Table 2).

**Table 2:** Sex distribution of study participants (n=40). Data have been presented as number of participants (n) and percentage.

Sex	Frequency (n)	Percentage
Female	1	2.5%
Male	39	97.5%
Total	40	100%

Side involvement

The study reveals a noteworthy imbalance in the affected shoulder among participants. A significant majority, comprising 70% (n=28) of the individuals, experienced shoulder instability in their right shoulder. This is in stark contrast to the involvement of the left shoulder, which only affected 30% (n=12) of the participants (Table 3).

**Table 3:** Side involvement of study participants (n=40) Data have been presented as number of participants (n) and percentage.

Side	Frequency (n)	Percentage
Left	12	30%
Right	28	70%
Total	40	100%

Mode of injury at the time of first dislocation

Road traffic accidents (RTA) were the most commonly reported cause of shoulder instability, accounting for 32.5% (n=13) of the cases. This was followed by Kabaddi injuries (17.5%, n=7) and volleyball injuries (12.5%, n=5). Other reported causes included sports activities like cricket (7.5%, n=3), high jump (5.0%, n=2), and overhead activities (7.5%, n=3). Interestingly, a few cases were attributed to seizure activity (5.0%, n=2) and electric shock (2.5%, n=1) (Table 4).

**Table 4:** Distribution of mode of injury at first dislocation (n=40) Data has been presented as number of participants (n) and percentage.

Mode of injury	Frequency (n)	Percentage
Cricket	3	7.5%
Electric shock	1	2.5%
High jump	2	5.0%
Kabaddi	7	17.5%
Kho kho	1	2.5%
Martial arts	1	2.5%
Overhead activity	3	7.5%
Road traffic accident (RTA)	13	32.5%
Seizure activity	2	5.0%
Swimming	2	5.0%
Volleyball	5	12.5%
Total	40	100.0%

Distribution of the number of prior dislocations

The distribution of prior shoulder dislocations among participants is varied. The number of prior dislocations ranged from 4 to 20 (Table 5).

**Table 5:** Distribution of number of prior dislocations (n=40). Data have been presented as number of participants (n) and percentage.

Number of prior dislocations	Frequency (n)	Percentage
4	4	10.0%
5	1	2.5%
6	4	10.0%
7	4	10.0%
8	7	17.5%
9	2	5.0%
10	6	15.0%
11	1	2.5%
12	3	7.5%
15	4	10.0%
20	4	10.0%
Total	40	100.0%

Number of suture anchors used

All 40 patients had Bankart lesions and underwent Arthroscopic Bankart repair. The majority of the Bankart repair procedures (87.5%, n=35) utilized two suture anchors. Three anchors were used in a smaller portion of cases (12.5%, n=5) (Table 6).

**Table 6:** Distribution of number of suture anchors used during Bankart repair in study participants (n=40). Data have been presented as number of participants (n) and percentage.

Number of anchors	Frequency (n)	Percentage
2	35	87.5%
3	5	12.5%
Total	40	100.0%

Remplissage in Hill-Sachs lesion

Remplissage was exclusively performed on participants with engaging Hill-Sachs lesions. A total of 22 participants (55%) fell into this category and all of them underwent the procedure. The remaining 45% of participants (18 individuals) had non-engaging Hill-Sachs lesions. None of these participants received a remplissage procedure (Table 7).

**Table 7:** Remplissage in Hill-Sachs lesion distribution. Data have been presented as participants (n) with remplissage procedure and without remplissage procedure and percentage (%).

Hill-Sachs lesion	Remplissage done	No remplissage	Total
Engaging	22	0	22 (55%)
Non-engaging	0	18	18 (45%)
Total	22	18	40 (100%)

Range of motion outcomes

There was a statistically significant improvement in flexion, abduction, external rotation in adduction, external rotation in abduction, and internal rotation at both three months and six months post-surgery compared to pre-operative measurements. No statistically significant change was observed in shoulder extension at any follow-up time point (Table 8).

**Table 8:** The pre-operative, three-month post-operative, and six-month post-operative follow-up measurements of range of motion in the shoulder joint for the study participants (n=40). *P-value <0.05 is considered significant. Data have been presented as mean, standard deviation (SD) between pre-operative and post-operative values, and p-value from paired t-tests.

Range of motion (degrees)	Pre-operative mean (SD)	3 months post-operative mean (SD)	6 months post-operative mean (SD)	p-Value (pre-operative vs. 3 months post-operative)	p-Value (pre-operative vs. 6 months post-operative)
Flexion (degrees)	153.75 (17.348)	163.63 (8.005)	171.88 (6.951)	<0.001	<0.001*
Extension (degrees)	40.00 (0.000)	40.00 (0.000)	40.00 (0.000)	-	-
External rotation in adduction (degrees)	50.00 (9.541)	65.00 (5.991)	69.75 (4.229)	<0.001	<0.001*
External rotation in abduction (degrees)	64.13 (12.398)	79.50 (7.321)	88.13 (4.762)	<0.001	<0.001*
Internal rotation (degrees)	51.88 (8.140)	67.00 (4.641)	70.00 (1.961)	<0.001	<0.001*

Remplissage and range of motion at six months

At six months post-surgery, there were no statistically significant differences in flexion, extension, abduction, or internal rotation between patients who received remplissage and those who did not. There was a statistically significant difference in external rotation in abduction at six months, with the participants that received remplissage (n=22, 55%) showing a mean external rotation of 86.59° compared to the participants without remplissage (n=18, 45%) of 90° (Table 9).

**Table 9:** Comparison of range of motion values at six months post-surgery in patients who underwent arthroscopic Bankart repair with (yes) and without (no) remplissage. *P-value <0.05 is considered significant. Data have been presented for each group (remplissage yes/no) as mean, standard deviation (SD), and p-value from unpaired t-tests.

Range of motion (degrees)	Remplissage	Mean (SD)	p-Value
Flexion (degrees)	Yes	171.36 (7.102)	0.613
	No	172.50 (6.913)	
Extension (degrees)	Yes	40.00 (0.000)	-
	No	40.00 (0.000)	
Abduction (degrees)	Yes	171.36 (7.102)	0.894
	No	171.67 (7.071)	
External rotation in adduction (degrees)	Yes	69.55 (2.132)	0.740
	No	70.00 (5.941)	
External rotation in abduction (degrees)	Yes	86.59 (4.194)	0.022*
	No	90.00 (4.851)	
Internal rotation (degrees)	Yes	70.00 (0.000)	1.000
	No	70.00 (2.970)	

Functional outcome scores after arthroscopic Bankart repair with and without remplissage

All three functional outcome scores (ASES, Rowe, and Quick DASH) showed statistically significant improvements at both three months and six months post-surgery compared to pre-operative scores (Table 10).

**Table 10:** Changes in functional outcome scores following surgery (n=40). *P-value <0.05 is considered significant. Data have been presented as mean, standard deviation (SD) between pre-operative and post-operative scores, and p-value from paired t-tests. ASES score: American Shoulder and Elbow Surgeons score; DASH score: Disabilities of the Arm, Shoulder, and Hand score

Outcome score	Pre-operative mean (SD)	3 months post-operative mean (SD)	6 months post-operative mean (SD)	p-Value (pre-operative vs. 3 months)	p-Value (pre-operative vs. 6 months)
ASES score	40.08 (8.87)	74.36 (8.11)	89.64 (3.00)	<0.001	<0.001*
Rowe score	17.13 (9.53)	76.63 (11.12)	93.63 (2.99)	<0.001	<0.001*
Quick DASH score	54.31 (10.44)	22.72 (5.72)	4.67 (0.61)	<0.001	<0.001*

Impact of number of anchors on functional outcomes at six months

There were no statistically significant differences in the ASES score, Rowe score, or Quick DASH score at six months post-surgery between patients who received two anchors (n=35, 87.5%) and those who received three anchors (n=5, 12.5%) during arthroscopic Bankart repair (Table 11).

**Table 11:** Comparision of functional scores in patients who received two anchors versus three anchors during arthroscopic Bankart repair. Data have been from unpaired t-tests presented for each group (number of anchors) as mean, standard deviation (SD), and p-value. ASES score: American Shoulder and Elbow Surgeons score; DASH score: Disabilities of the Arm, Shoulder, and Hand score

Outcome score	Number of anchors	Mean (SD)	p-Value
ASES score (6 months)	2	89.97 (3.02)	0.060
	3	87.28 (1.52)	
Rowe score (6 months)	2	93.57 (2.86)	0.769
	3	94.00 (4.18)	
Quick DASH score (6 months)	2	4.69 (0.65)	0.509
	3	4.50 (0.00)	

Impact of remplissage on functional outcomes at six months

There were no statistically significant differences in the ASES score, Rowe score, or Quick DASH score at six months post-surgery between patients who received remplissage and those who did not (Table 12).

**Table 12:** Comparison of functional outcome scores at six months post-operatively in patients who underwent arthroscopic Bankart repair with and without remplissage. Data have been presented from unpaired t-tests for each group (remplissage yes/no) as mean, standard deviation (SD), and p-value. ASES score: American Shoulder and Elbow Surgeons score; DASH score: Disabilities of the Arm, Shoulder, and Hand score

Outcome score	Remplissage	Mean (SD)	p-Value
ASES score (6 months post-operative)	Yes	89.02 (2.39)	0.173
	No	90.39 (3.54)	
Rowe score (6 months post-operative)	Yes	93.86 (2.64)	0.584
	No	93.33 (3.43)	
Quick DASH score (6 months post-operative)	Yes	4.61 (0.49)	0.446
	No	4.76 (0.74)	

## Discussion

Anterior shoulder instability is the most common form of glenohumeral instability [1]. Shoulder instability most commonly affects people who are in their late teens to mid-30s as reported by Hovelius [2]. Similarly, in our study, 50% were in the age group of 19-25 years, 30% in 26-35 years, and the remaining 20% in 36-50 years. Nearly all our patients were males (39 out of 40) as they were involved in sports and road traffic accidents. The right shoulder was involved in 28 patients (70%).

Trauma was the leading cause of instability in our study, with road traffic accidents (RTA) being the most prevalent (32.5%). This is in line with the 95.6% trauma-related rate reported by Rowe and Zarins in their study of 500 patients [16]. Various sports injuries followed in prevalence as follows: Kabaddi (17.5%), volleyball (12.5%), cricket (7.5%), and high jump (5.0%). Interestingly, a small number of cases were attributed to seizure activity (5.0%) and electric shock (2.5%).

All patients underwent Bankart repair during surgery, with the majority (87.5%) receiving two suture anchors. This finding supports Ballal et al.'s suggestion that the number and placement of suture anchors are crucial for a successful repair [17]. Engaging Hill-Sachs lesions were present in 55% of patients. Notably, remplissage procedure was performed in patients with engaging Hill-Sachs lesions.

Our results demonstrate significant improvements in all measured aspects of range of motion (flexion, abduction, external rotation in both adduction and abduction, and internal rotation) following surgery. These findings are consistent with previous research by Mishra et al. who reported a good range of motion after arthroscopic Bankart repair [18]. Interestingly, patients who underwent remplissage exhibited a slightly lower average (86.59°) external rotation in abduction compared to those who received Bankart repair alone (90°) which is consistent with the results of Cho et al. [19]. However, this trade-off may be acceptable considering the potential benefit of remplissage in preventing recurrence.

Functional outcome scores (ASES, ROWE, and Quick DASH scores) showed significant improvement post-operatively. Additionally, there was no statistically significant difference in scores between patients who received two vs. three suture anchors for Bankart repair. This aligns with the work of Yan et al. who concluded that the type, number, and presence of a bony Bankart lesion may not significantly impact functional outcomes [12]. These findings suggest that meticulous surgical technique, proper patient selection, and optimal anchor placement are likely more important factors for functional recovery than the specific number of anchors used.

Studies investigating arthroscopic Bankart repair for anterior shoulder instability report a wide range of recurrence rates, from as low as 2.5% to as high as 30% [20]. This variability highlights the potential influence of various factors on long-term outcomes. Ide et al. documented a 7% failure rate in young, athletic patients undergoing arthroscopic Bankart repair [21]. In contrast, Mishra et al. observed no dislocations following the procedure in their 2012 study [18]. Our study aligns with these findings, with a single patient (2.5%) experiencing a recurrence after Bankart repair alone. This patient subsequently underwent an open Latarjet procedure for definitive stabilization.

Further research with larger patient cohorts and longer follow-up periods could provide more robust data on long-term outcomes and potential risk factors for recurrence. Additionally, investigating the cost-effectiveness of using two suture anchors compared to a higher number warrants further exploration.

## Conclusions

Our study emphasizes the importance of proper capsulolabral tissue elevation for achieving a sufficient labral bump during Bankart repair. This technique allowed us to efficiently use only two suture anchors in 35 cases and potentially reduced cost burden without compromising outcomes. Additionally, remplissage was performed on all identified engaging Hill-Sachs lesions. We found that proper anchor placement and suturing technique are crucial for successful Bankart repair.

Overall, this study adds to the growing body of evidence supporting arthroscopic Bankart repair as a successful treatment for anterior shoulder instability, particularly in young males. The emphasis on proper surgical technique and the potential cost benefits of a two-anchor approach are valuable contributions to the field.
